# Systematic Synergy of Glucose and GLP-1 to Stimulate Insulin Secretion Revealed by Quantitative Phosphoproteomics

**DOI:** 10.1038/s41598-017-00841-1

**Published:** 2017-04-21

**Authors:** Jia-shu Tang, Qing-run Li, Jia-ming Li, Jia-rui Wu, Rong Zeng

**Affiliations:** 1 Key Laboratory of Systems Biology, Institute of Biochemistry and Cell Biology, Shanghai Institutes for Biological Sciences, Chinese Academy of Sciences, 320 Yue-Yang Road, Shanghai, 200031 China; 2 Department of Life Sciences, ShanghaiTech University, 99 Haike Road, Shanghai, 201210 China

## Abstract

GLP-1 synergizes with glucose in regulating pancreatic β-cell function, including facilitating β-cell survival and insulin secretion. Though it has been widely accepted that phosphorylation is extremely important in regulating β-cell functions, our knowledge to the global mechanism is still limited. Here we performed a quantitative phosphoproteomics study to systematically present the synergistic regulation of INS-1E cell phosphoproteome mediated by glucose and GLP-1. We generated the largest pancreatic β-cell phosphoproteome by identifying 25,327 accurately localized phosphorylation sites on 5,389 proteins. Our results discovered several novel kinases regulated by glucose, GLP-1 or their synergism, and some of these kinases might act as downstream molecules of GLP-1 mediated PKA signaling cascade. A few phosphosites were regulated by both GLP-1 and glucose alone, and these target proteins were highly related to their biological function on pancreatic β-cells. Finally, we found glucose and GLP-1 executed their synergistic effect at multiple levels, especially at pathway level. Both GLP-1 and glucose participated in regulating every single step of the secretion pathway, and systematically synergized their effects in inducing insulin secretion.

## Introduction

Diabetes mellitus (DM), commonly referred to as diabetes, is a group of metabolic diseases characterized by high blood sugar levels over a prolonged period. About 8.3% of the adult population suffer these syndromes, of which type 2 diabetes makes up about 90% of the cases^1^. Impaired insulin secretion caused by pancreatic β-cell dysfunction is considered as one of the major factors associated with both type 1 and type 2 diabetes mellitus.

Several physiological secretagogues, such as glucose and incretin hormones can induce insulin secretion to maintain blood glucose homeostasis. Glucose induces insulin secretion in a two phase manner, including a rapidly initiated and transient first phase for a few minutes followed by a sustained second phase^2^. It has been commonly accepted that the fusion events conducted by the immediately releasable pool of predocked secretory granules and the mobilization of the reserve pool are the major mechanism of acute and sustained phase of glucose stimulated insulin secretion (GSIS) respectively^2^. Glucagon-like peptide-1 (GLP-1) is a 30 amino acid incretin hormone produced by the intestinal enteroendocrine L-cells^3^. It shares several common biological functions on pancreatic β-cells with glucose, such as promoting insulin secretion and cell survival^4^. GLP-1 can potentiate both acute and sustained phase of GSIS, and its insulinotropic function is active only at glucose concentrations above a certain level^5^. Besides potentiation of GSIS, GLP-1 also synergizes with glucose to regulate intracellular signaling in pancreatic β-cells, such as increasing [Ca^2+^]i^6^, inducing gene expression^7^, and regulating kinase activity^8^.

Phosphorylation is a crucial post-translational modification that regulates β-cell biological functions^9^. Several kinases, like Ca^2+^/calmodulin-dependent protein kinase II (CaMKII)^10^, 5′-AMP-activated protein kinase (AMPK)^11^, and protein kinase D (PKD)^12^ are involved in regulating GSIS. GLP-1 is reported to increase intracellular second messenger cAMP through activation of adenylyl cyclase^13^. cAMP signaling mediates the effect of GLP-1 in potentiating insulin secretion, through both PKA-dependent and PKA-independent mechanism^13^. Besides PKA, several other kinases, such as extracellular signal-regulated kinase (ERK), protein kinase B (Akt) were also reported to be activated by the treatment of GLP-1^8^, though their function was less focused. The exocytosis of insulin secretory granules (ISGs) is mediated by exocytotic apparatus proteins, such as soluble NH_2_-ethylmaleide-sensitive fusion protein attachment protein receptors (SNAREs)^14^. Though plenty of components of the exocytotic apparatus have been characterized, their phosphorylation regulation mediated by glucose, incretin hormones or their synergism is largely unknown.

Mass spectrometry-based quantitative proteomics now allow us to study the expression and modification of proteins at systems biology level^15^. Recently, several proteomics-based studies have been performed on pancreatic β-cells to elucidate the molecular mechanism of glucose homeostasis regulation and the pathogenesis of diabetes mellitus^16–19^, but most of these studies focused on proteome profiling and changes of protein expression in β-cells. Han *et al*. characterized the phosphoproteome of INS-1 cells, and identified 2,467 phosphorylation sites on 1,419 phosphoproteins^20^, but they failed to characterize the phosphorylation regulation related to β-cell function. In this study, stable isotope dimethyl labeling-based quantitative phosphoproteomics were applied to study the common, different and synergistic regulation of INS-1E cell (a well characterized rat insulinoma pancreatic beta-cell line) phosphoproteome mediated by glucose and GLP-1. Besides, we also characterized the phosphorylation dynamics during the process of glucose stimulated insulin secretion. Our data generated the largest INS-1E cell phosphoproteome through identifying 25,327 accurately localized phosphorylation sites on 5,389 proteins, and revealed that glucose and GLP-1 synergistically regulated the β-cell phosphoproteome during the process of insulin secretion at multiple levels.

## Results

### Quantification of INS-1E cell phosphoproteome

**Figure 1:**
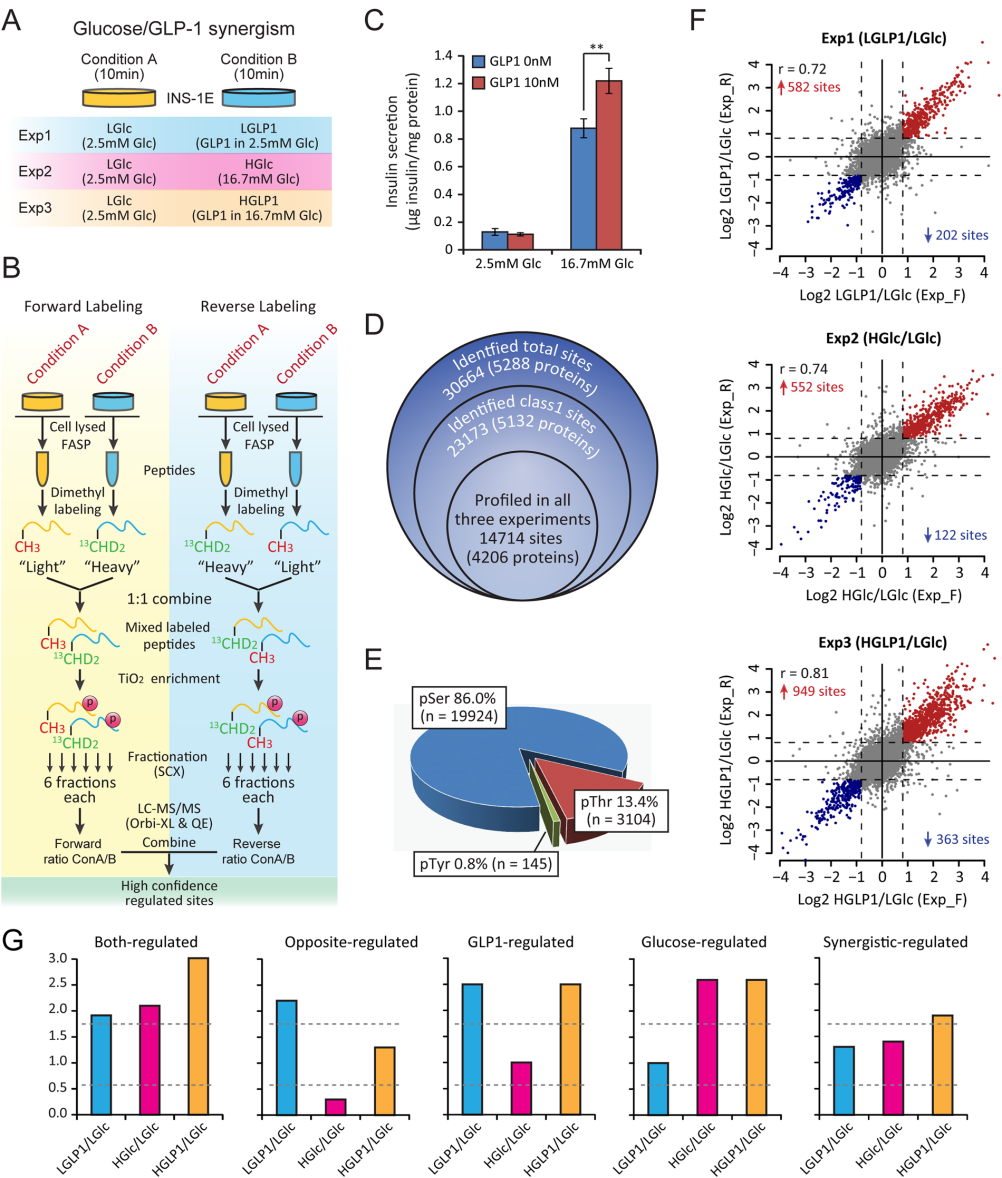
Quantification of the INS-1E cell phosphoproteome regulated by physiological secretagogues using tandem mass spectrometry. (**A**) Experimental design of the glucose/GLP-1 synergism experiment. Three experiments were contained in this set of experiments (Exp1 n = 4; Exp2 n = 4; Exp3 n = 4). (**B**) Workflow of the stable isotope dimethyl labeling-based quantitative phosphoproteomic analysis. **(C)** GLP-1 potentiates insulin secretion in a glucose dependent manner (**p < 0.01, Student’s t-test, n = 4, error bar: SEM). **(D)** Summary of identified and quantified phosphoproteome. **(E)** Distribution of accurately localized phosphorylated serine, threonine, and tyrosine residues in the combined data sets of all three experiments. **(F)** High-confidence regulated sites defined in all three experiments. Ratio condition B/A of each class 1 phosphorylation site determined in both forward and reverse labeling replicates are plotted. Down- or up-regulated phosphorylation sites are highlighted with blue and red spots respectively. Dashed line indicates 1.75-fold change threshold. Exp_F is short for forward labeling experiment, Exp_R is short for reversed labeling experiment. **(G)** Definition of phosphorylation regulation pattern mediated by GLP-1 (LGLP1), glucose (HGlc) and their synergism (HGLP1). The regulation pattern was divided into 5 classes. Dashed gray line indicates 1.75 fold change threshold.

GLP-1 involves in several biological effects on pancreatic β-cells with glucose, including potentiating insulin secretion in a glucose dependent manner (Fig. 1C) and increasing β-cell mass as well^4^. In order to study the common, different and synergistic effect of glucose and GLP-1 on β-cells systematically, a stable isotope dimethyl labeling-based quantitative phosphoproteomics strategy was applied as displayed in Fig. 1A and B, composed of three experiments. In Exp1, INS-1E cells treated with low glucose (LGlc) were compared to INS-1E cells treated with GLP-1 alone (LGLP1). In Exp2, low glucose treatment (LGlc) was compared to high glucose treatment (HGlc), and in Exp3, low glucose treatment (LGlc) was compared to the combination of GLP-1 and high glucose (HGLP1) (Fig. 1A). A forward-reverse labeling strategy was used to screen the phosphorylation sites regulated by these secretagogues with high confidence, and biological duplicates were analysed in both forward and reverse labeling experiments (Fig. 1B). In each replicate experiment, phosphopeptides enriched by TiO_2_ were fractionated by strong cation exchange (SCX) and analysed using collision-induced dissociation (CID) mode on LTQ-Orbitrap XL or higher energy collisional dissociation (HCD) mode on Q-Exactive respectively to ensure deep sequencing of INS-1E phosphoproteome (Fig. 1B). Three groups of experiments with four biological replicas each finally generated 72 raw files, resulting the identification of 30,664 phosphorylation sites on 5,288 proteins (Fig. 1D, Supplementary Table S1), with high quality of MS/MS identification (Supplementary Fig. S1A and B).

Using commonly accepted criterion (localization probability > 0.75, score diff > 5)^21^, 23,173 phosphorylation sites on 5,132 proteins could be accurately localized (class 1 sites, median localization probability for all class 1 sites was 0.999, Supplementary Fig. S1C and D), including 19,924 phosphorylated serine (pSer) residues, 3,104 pThr, and 145 pTyr sites (Fig. 1E). We classified all proteins with class 1 sites according to “molecular function” annotated by Gene Ontology (GO) database and found they spanned a broad range of categories (Supplementary Fig. S1E). For example, we found 3,739 phosphorylation sites on 861 transcription regulators, 1,228 sites on 200 GTPase regulators, which were likely to be related to β-cell function. Not only did we achieve high identification coverage of β-cell phosphoproteome, but also with reliable quantification information in this data set, as indicated by the good reproducibility of quantification among replicates (Supplementary Fig. S2A).

### β-cell phosphoproteome regulated by physiological secretagogues

We chose phosphopeptides with the least number of phosphorylation sites (Nmods in MaxQaunt) to quantify the contained sites for each treatment experiment which was the default option of MaxQuant (Supplementary Table S1), and 14,714 phosphorylation sites on 4,206 proteins could be profiled across all three treatment experiments if they shared the same Nmods in quantifying the same phosphorylation site (Fig. 1D). Log_2_-transformed ratio of treatment/basal condition (condition B/A in Fig. 1A) in both forward and reverse labeling replicates of all three experiments exhibited a narrow Gaussian distribution around 0, indicating both GLP-1 (LGLP1) and glucose (HGlc), as well as their cooperation (HGLP1) only moderately modified the INS-1E cell phosphoproteome (Supplementary Fig. S2B). Fold change threshold was determined by boxplot algorithm to the log_2_-transformed data (rough equating to a 1.75-fold change in this data set) (Supplementary Fig. S2B), and high-confidence regulated sites were determined if the ratio of condition B/A reached the fold change threshold in both forward and reverse labeling replicates (see Methods for details). Thus, 784 class 1 sites on 576 proteins were regulated by GLP-1 (LGLP1), 674 class 1 sites on 462 proteins were regulated by glucose (HGlc) and 1312 class 1 sites on 793 proteins were regulated by their synergism (HGLP1) with high confidence (Fig. 1F). We also defined a class of medium-confidence regulated sites to facilitate the comparison among three treatments (Supplementary Fig. S2C). For phosphorylation sites that was quantified in forward or reverse labeling experiment only, the one with ratio achieved 1.75-fold change threshold in the quantified experiment was defined as medium-confidence regulated sites, and for sites quantified in both labeling replicates, the one with ratio achieved 1.75-fold change in one labeling replicate and achieved 1.5-fold in the other was also termed medium-confidence regulated sites (Supplementary Fig. S2C, see Methods for details). Using this criterion, we generated the class of medium-confidence regulated sites about the same size as high-confidence ones (Supplementary Fig. S2C, Supplementary Table S2). The phosphorylation regulation pattern mediated by GLP-1, glucose and their synergism could be complex, we defined the different regulation patterns in Fig. 1G. The phosphorylation change mediated by the cooperation of glucose and GLP-1 (HGLP1) could be the result of either of these treatments alone (GLP1-regulated and Glucose-regulated in Fig. 1G), or glucose (HGlc) and GLP-1 (LGLP1) could regulate the same target site either in identical or opposite tendency (Both-regulated and Opposite-regulated in Fig. 1G). It was also possible that though treatment of the GLP-1 (LGLP1) and glucose (HGlc) alone did not significantly modify the phosphorylation status of a site, but their cooperation (HGLP1) could reach the regulation threshold (Synergistic-regulated in Fig. 1G).

### Kinase-substrate prediction

**Figure 2:**
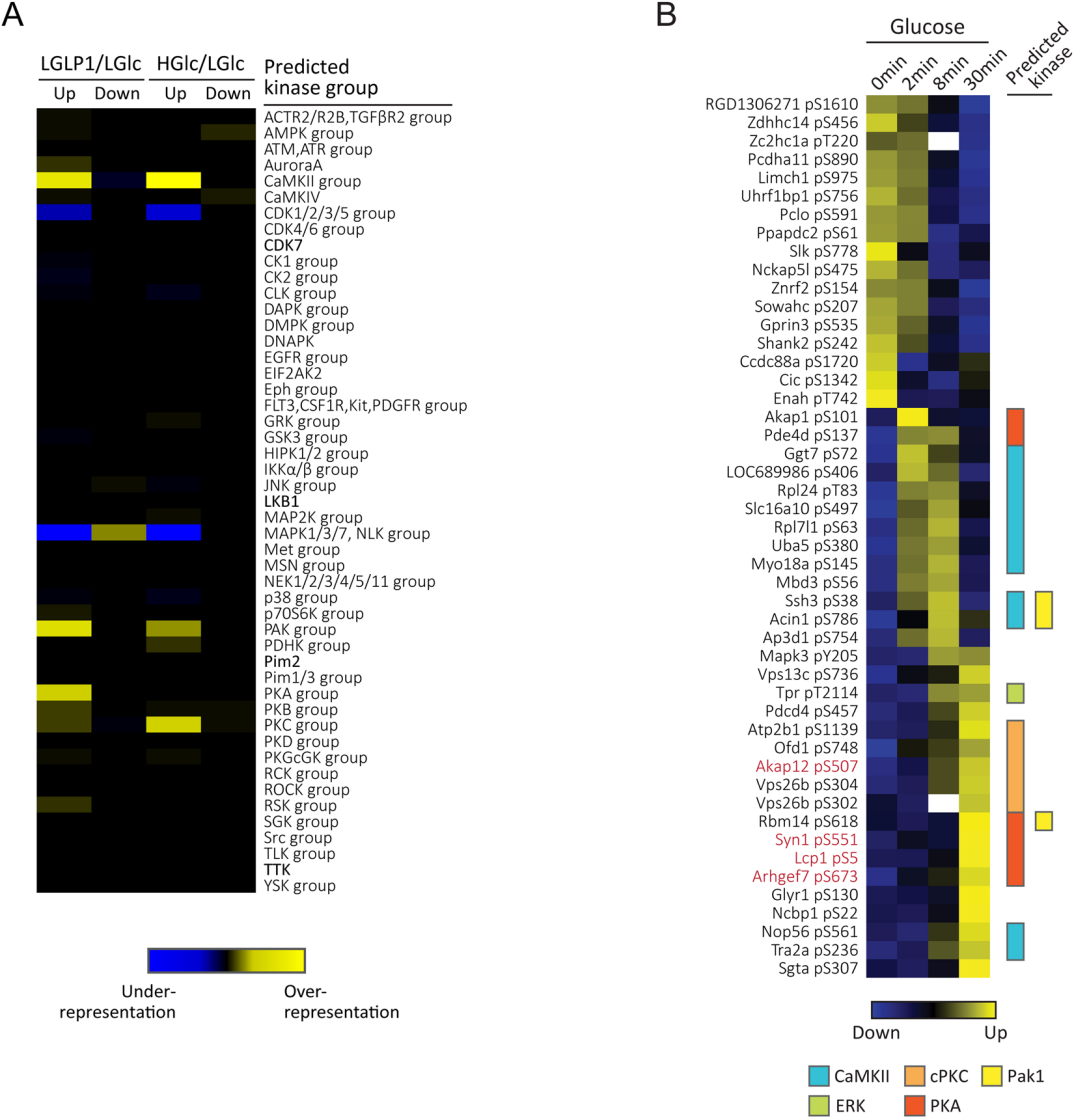
Kinase-substrate prediction. **(A)** Heat map of secretagogue regulated kinase substrates. Kinase substrate prediction was performed using NetworKIN 3.0. High confidence regulated sites (up and down-regulated) were compared to quantified phosphorylation sites for kinase group substrates enrichment. The heat map shows over- (yellow) and underrepresentation (blue) of predicted kinase substrates among regulated sites. **(B)** Heatmap showing the glucose treatment temporal profiles of phosphorylation sites regulated by both GLP-1 and glucose. Only phosphorylation sites regulated by both GLP-1 in the glucose/GLP-1 synergism experiment (Exp1 in Fig. 1A) and by glucose in the glucose time course experiment (Supplementary Fig. S3A) were plotted. Kinases predicted by NetworKIN algorithm of each site are listed on the right side. Characters in red represent the reported kinase-substrate relationship.

We predicted possible kinase-substrate relationships for all class 1 sites with NetworKIN algorithm^22^ and explored the kinase substrates overrepresented among the phosphorylation sites regulated by GLP-1 or glucose. We found substrates of several kinases were overrepresented in both GLP-1 regulated sites and glucose regulated sites, such as substrates of CaMKII and p21-activated kinase (PAK) family (Fig. 2A), in agreement with their functions reported in the process of insulin secretion^10, 23^. As expected, PKA substrates were significantly enriched among GLP-1 positively regulated sites, but surprisingly we did not observe the same enrichment of PKA substrates among glucose regulated sites (Fig. 2A), though the cAMP/PKA signaling is one of the major mechanism of insulin exocytosis mediated by both GLP-1 and glucose^24^. It was possible that longer treatment of glucose was required for PKA activation, as the cAMP generation induced by glucose alone was less active than by GLP-1^25^. In order to figure out whether this was the case, a set of glucose time course experiments was performed using a spike-in stable isotope labeling by amino acids in cell culture (SILAC) strategy for quantitative phosphoproteomics studies (Supplementary Fig. S3A). Glucose treatment of 0 min, 2 min, 8 min and 30 min was selected representing the basal condition, the apex of acute phase, the end of acute phase and the sustained phase of insulin secretion (Supplementary Fig. S3A). Three biological replicates were performed for each time point experiment with one replicate using TiO_2_ + SAX (strong anion exchange) and two replicates using multiple TiO_2_ incubation strategy for phosphopeptides enrichment and fractionation. This set of experiments led to the identification of 8,618 accurately localized phosphorylation sites on 2,697 proteins, and 6,554 sites on 2,351 proteins could be profiled across four glucose treatment time points (Supplementary Fig. S3B, Supplementary Table S3). Using 1.5-fold change threshold determined by boxplot (see Supplementary methods for details), 664 class 1 sites on 468 proteins were regulated by glucose during the process of insulin secretion (Supplementary Fig. S3D, Supplementary Table S4), and PKA was indeed activated only upon 30 min treatment of glucose as indicated by its own activation site (Supplementary Fig. S3E). We then compared phosphorylation sites regulated by LGLP1 (Exp1 in Fig. 1A) with phosphorylation sites regulated by glucose in the glucose time course experiment (Supplementary Fig. S3A), and found 48 sites were regulated by both GLP-1 and glucose along the period of treatment (Fig. 2B, Supplementary Table S5). Among these both-regulated sites, a slight enrichment of PKA substrates was observed (*P* value 0.00139, Hypergeometric test, n = 6), and most of the PKA substrates were indeed phosphorylated upon prolonged treatment of glucose (Fig. 2B).

### Synergistic effect mediated by GLP-1 and glucose at multiple levels

**Figure 3:**
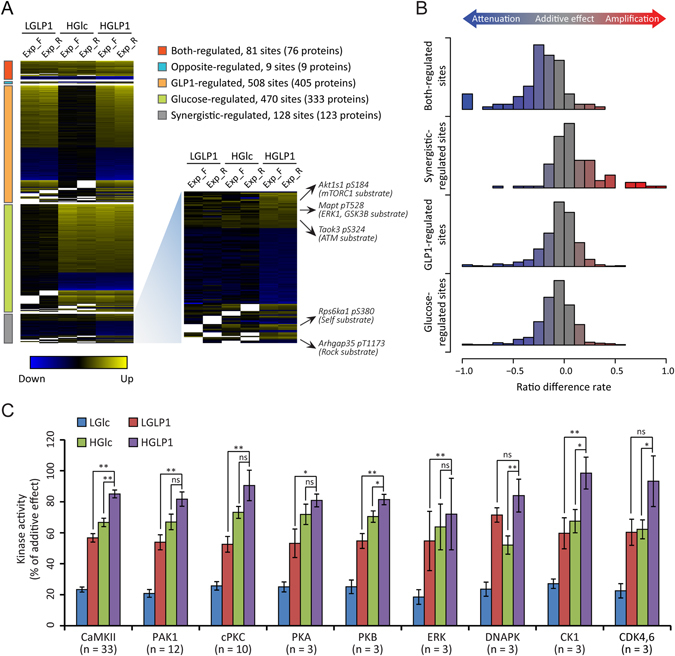
Synergistic regulation of β-cell phosphoproteome mediated by GLP-1 and glucose at site level. **(A)** Heatmap showing the ratio condition B/A in three experiments for class 1 sites regulated by HGLP1 with high confidence. These phosphorylation sites were classified into 5 categories as defined in Fig. 1G. Synergistic-regulated sites with their biological functions reported are listed, together with their reported kinases. **(B)** Histogram of the ratio difference rate occurrences showing whether GLP-1 and glucose induced phosphorylation change of each site in an additive manner. All HGLP1 high confidence regulated sites were used for calculation. Ratio difference rate from additive effect was defined in Supplementary Fig. S4. (**C**) Activation of kinases mediated by GLP-1 (LGLP1), glucose (HGlc) or their synergism (HGLP1) according to the phosphorylation status of their substrates predicted by NetworKIN algorithm. Only both-regulated sites mediated by GLP-1 and glucose were used for kinase activation calculation. Phosphorylation status of each site according to each treatment were divided by ratio_additive as defined in Supplemental Fig. S4. Paired Student’s t-test for each site was applied to calculate the significance of activation status of their predicted kinase. ns, non-significant. *p < 0.05; **p < 0.01 (paired Student’s t test). Data were represented in mean ± SEM. n stands for the number of predicted substrates of each kinase.

We classified GLP-1 plus high glucose (HGLP1) high-confidence regulated sites into 5 categories according to the phosphorylation regulation pattern defined in Fig. 1G (Fig. 3A). As expected, most of the phosphorylation regulation mediated by the cooperation of GLP-1 plus glucose (HGLP1) were caused by either of these treatments alone (GLP-1 regulated and glucose-regulated in Fig. 1G). Interestingly, we found a small part of phosphorylation sites (128 class 1 sites on 123 proteins) termed as synergistic-regulated sites (Fig. 3A). According to the phosphorylation sites with their upstream kinase reported, no specific kinase was likely to answer for this synergistic effect at site level (Fig. 3A). In order to figure out whether the synergistic regulation at site level was caused by additive effect mediated by GLP-1 and glucose, we defined the ratio_additive to represent the additive effect of phosphorylation change for every single site mediated by GLP-1 and glucose (Supplementary Fig. S4), and compared this value with the ratio HGLP1/LGlc (Fig. 3B, Supplementary Table S6). Not surprisingly, for most phosphorylation sites, the ratio_additive was very close to the ratio HGLP1/LGlc, suggested an additive regulation at site level mediated by GLP-1 and glucose. Though additive regulation was the case for most phosphorylation sites, we found for the majority of both-regulated sites, co-administration of these two secretagogues did not achieve the additive level in phosphorylation change (Fig. 3B, Supplementary Fig. S5A). Among these both-regulated sites, GLP-1 and glucose executed their synergistic effect on negatively regulated ones more likely in a multiplicative manner (Supplementary Fig. S5C). Though co-administration of GLP-1 and glucose (HGLP1) induced more substantial phosphorylation change for most of the both-regulated sites than either of the treatment alone (Supplementary Fig. S5B), synergistic effect at site level mediated by them did not reach either additive or multiplicative-level (Supplementary Fig. S5A and C). We then attempted to figure out the synergistic effect on kinase activity mediated by GLP-1 and glucose based on the phosphorylation change of their substrate sites predicted by NetworKIN algorithm (Fig. 3C, Supplementary Table S6). PAK1, PKA and ERK kinase group were activated by both GLP-1 (LGLP1) and glucose (HGlc), but the co-administration of GLP-1 did not significantly potentiate glucose induced activation of these kinases, as previously reported^8^. The co-administration of GLP-1 and glucose (HGLP1) activated CaMKII and Akt more significantly than either of the treatment alone, but still their synergism did not achieve additive level in activating these two kinases.

**Figure 4:**
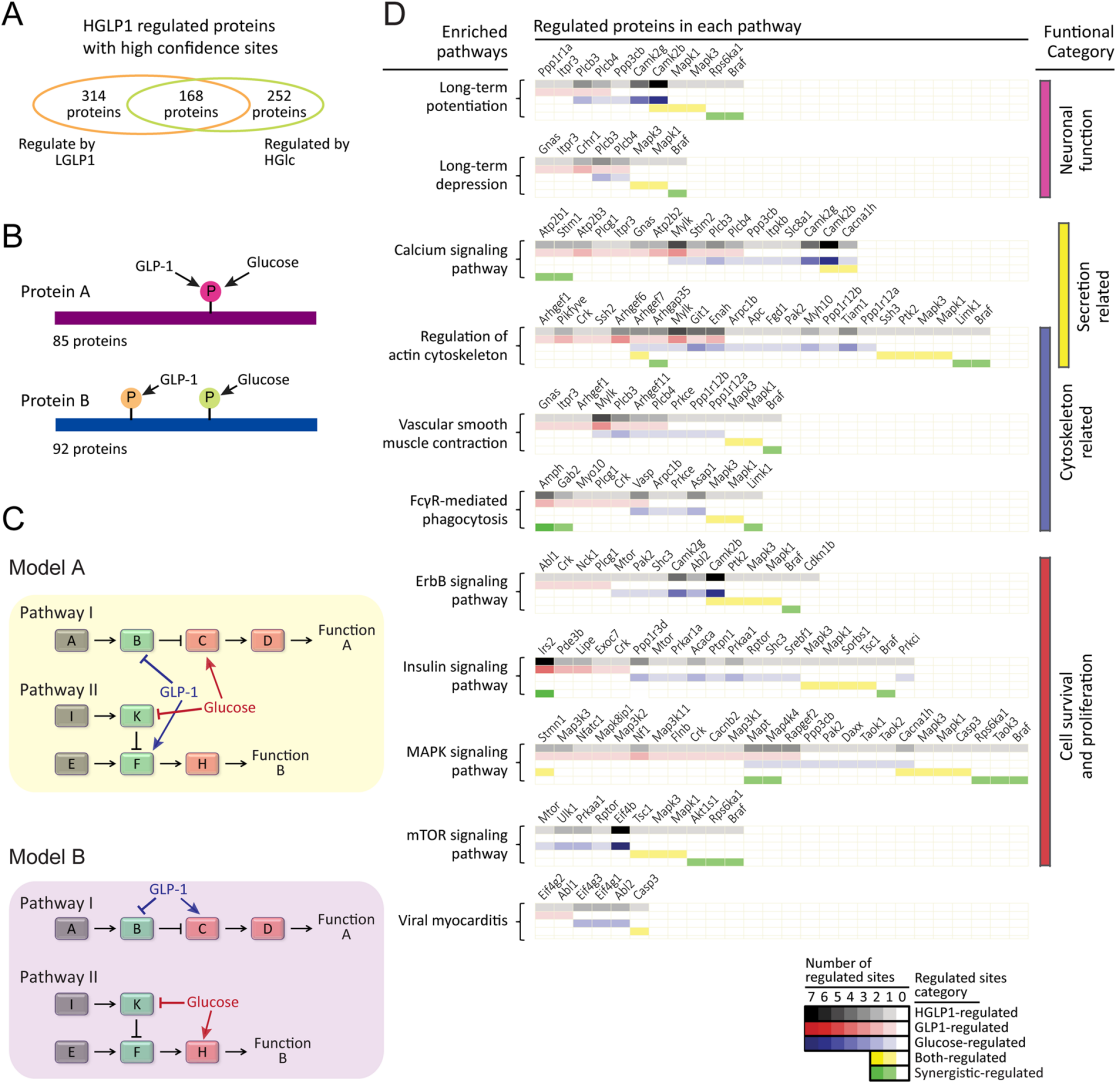
Synergistic effect at protein and pathway level. (**A**) Venn-diagram showing overlap of GLP-1 (LGLP1) and glucose (HGlc) regulated phosphoproteins. Only proteins with phosphorylation sites regulated by HGLP1 with high-confidence were taken into count. **(B)** Diagram representing different regulation pattern on the same protein mediated by GLP-1 and glucose. GLP-1 and glucose could regulated the phosphorylation status of identical or distinct sites of the same protein. (**C)** Ideal models of pathway level synergism. Model A means GLP-1 and glucose regulate different protein targets in the same pathway. Model B means GLP-1 and glucose choose to regulate the distinct pathways. The rectangles stand for proteins involved in the pathway. **(D)** Heatmap representing the number of phosphorylation sites with different regulation patterns (defined in Fig. 1G) on proteins in enriched KEGG pathways. KEGG pathway enrichment analysis was performed using DAVID algorithm with default parameters by comparing proteins possessing HGLP1 high-confidence regulated sites with proteins possessing quantified sites.

There was a significant tendency that GLP-1 (LGLP1) and glucose (HGlc) regulate the phosphorylation status of the same proteins (Supplementary Fig. S6A), either through the identical sites or distinct sites. It is often the case that multiple sites should be phosphorylated (or dephosphorylated) for a protein to completely execute their function, this can be progressed concurrently or hierarchically, catalyzed by one kinase or involved two or more distinct kinases^26^. Among the 799 phosphoproteins with phosphorylation sites regulated by HGLP1 with high confidence, 168 proteins were regulated by both GLP-1 and glucose (Fig. 4A), and 92 of these proteins were regulated at different sites by these two secretagogues (Fig. 4B, Supplementary Fig. S6B). These phosphoproteins covered a large variety of molecular functions, including protein kinases, GTPase regulators, transcription regulators as well as ion channels (Supplementary Fig. S6B). These proteins might be targets of synergistic effect mediated by GLP-1 and glucose at protein level, possibly related to β-cell functions.

It was also likely that GLP-1 and glucose could regulate the same pathway through different targets or they could regulate the distinct pathways and finally achieved the synergistic effect on β-cell functions at the pathway level (Fig. 4C). In order to find out which really happened, we first screened the enriched pathways regulated by the cooperation of GLP-1 and glucose (HGLP1), then the regulation pattern of each site on each protein within the enriched pathways were calculated (Fig. 4D). According to DAVID algorithm, 11 pathways were over-represented among HGLP1 regulated phosphoproteins, most of which were highly related to their effect on β-cell functions, like regulation of secretion, cytoskeleton organization and cell survival. Phosphoproteins regulated by glucose alone (HGlc) were also enriched in several of these pathways (Supplementary Fig. S7), such as “calcium signaling pathways” and “MAPK signaling pathway”. Co-administration of GLP-1 extended the regulated targets in these pathways (Fig. 4D), and caused increased number of enriched pathways (Supplementary Fig. S7). Both-regulated and synergistic-regulated phosphorylation sites (defined in Fig. 1G) were partly involved in these enriched pathways. For most pathways, phosphoproteins regulated by GLP-1 (LGLP1) or glucose (HGlc) alone shared similar population (Fig. 4D), which meant that GLP-1 and glucose preferred to regulate the same pathway but through several different targets to execute their synergistic effects on β-cell functions at pathway level.

### Secretion pathway synergistically regulated by GLP-1 and glucose

**Figure 5:**
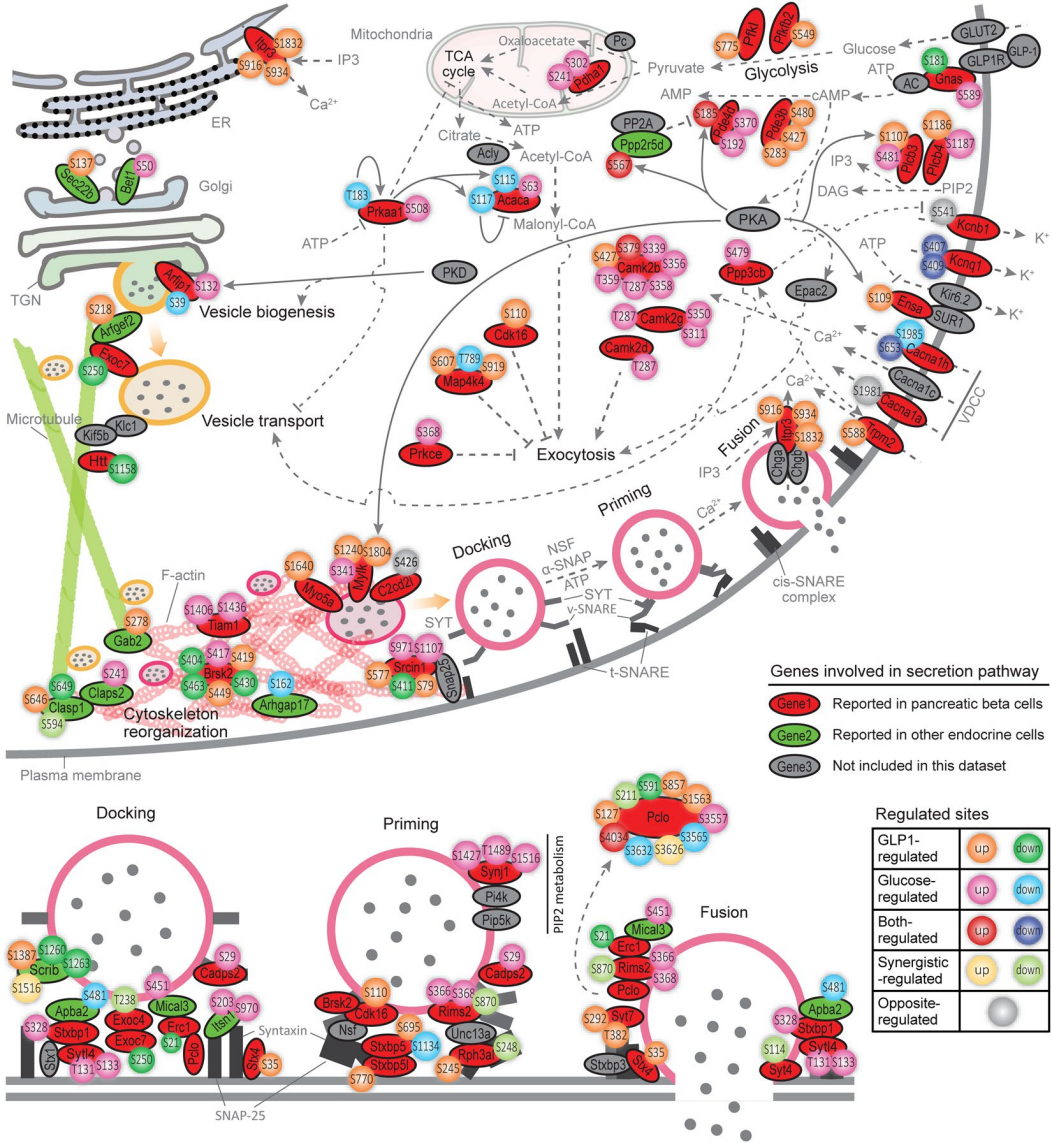
Synergistic regulation of secretion pathway. Regulated proteins annotated by GO database matching the biological process terms “insulin secretion” or “exocytosis” were presented to clarify the synergistic effect mediated by glucose and GLP-1 in secretion pathway. Only proteins with phosphorylation sites regulated by GLP-1 (LGLP1), glucose (HGlc) or their synergism (HGLP1) with high confidence were taken into count. The regulated sites were color-coded according to different regulation pattern as defined in Fig. 1G. Sold line means reported kinase-substrate relationship.

Nowadays plenty of proteins participated in exocytosis pathways have been characterized in pancreatic beta cells or other endocrine cells, such as exocyst complex proteins^27^ and SNAREs^14^. We then focused on the secretion pathway to present the mechanism of synergistic action on insulin secretion mediated by GLP-1 and glucose. 59 secretion pathway related proteins were phosphorylation regulated by GLP1 (LGLP1), glucose (HGlc) or their synergism (HGLP1) according to our data, including proteins involved in delivering extracellular signals (such as kinases and second messenger regulators) and effector proteins functioned directly in the exocytosis process (Fig. 5, Supplementary Table S7). These two secretagogues seemed to regulate nearly every step of exocytosis pathway, including proximal steps such as vesicle biogenesis, transport and distal ends like vesicle docking, priming, fusion as well as cytoskeleton reorganization.

Several target sites were regulated by GLP-1 and glucose together (both-regulated sites). For example, both GLP-1 and glucose participated in the regulation of intracellular cAMP homeostasis via not only activating adenylyl cyclase but also regulating the hydrolysis of this second message, through phosphorylating the same PKA target sites on protein phosphatase 2A regulatory subunit B56δ (*Ppp2r5d*) and cAMP-specific phosphodiesterase PDE4D (*Pde4d*) (Fig. 5)^28, 29^. Besides, both treatment of GLP-1 (LGLP1) and glucose (HGlc) decreased the phosphorylation level of Ser409 on voltage-gated potassium channel KvLQT1 (*Kcnq1*) (Fig. 5), thus reduced the outward K^+^ currents through this ion channel, contributed to the maintenance of depolarization status of cell membrane^30^. Interestingly, we also noticed that several phosphorylation sites were oppositely regulated by GLP-1 and glucose (opposite-regulated sites, colored gray in Fig. 5), though the amount of this type of sites was limited. For example, glucose induced the de-phosphorylation of Ca^2+^-dependent protein phosphatase calcineurin target site on voltage-gated potassium channel Kv2.1 (*Kcnb1*), which would activate this K^+^ channel and increase outward K^+^ currents thus impaired insulin exocytosis^31^. While treatment of GLP-1 alone (LGLP1) potentiated the phosphorylation level of this site, thus maintained this site in an unregulated manner and kept the depolarization status of cell membrane by cooperation of these two secretagogues.

As described above, GLP-1 and glucose preferred to regulate different targets within the same pathway to execute their synergistic effects at pathway level. They also shared similar amount of regulated proteins in the secretion pathway (37 proteins regulated by GLP-1 and 37 proteins by glucose), and most of these proteins were regulated by GLP-1 or glucose only (Fig. 5). For example, GLP-1 itself facilitated vesicle transport along cortical actin cytoskeleton through phosphorylating Ser1640 on myosin-Va^32^; while glucose alone could mediate vesicle biogenesis through phosphorylating the PKD substrate site (Ser132) on Arfaptin-1^33^. At more detailed level, even every single step of the exocytotic process, such as vesicle transport, docking, priming and fusion were also regulated by GLP-1 and glucose synergistically, mainly through distinct target proteins. Both GLP-1 and glucose seemed to take part in regulation of glucose metabolism (Fig. 5). Enzymes involved in glycolysis were mainly regulated by GLP-1 (LGLP1), while glucose (HGlc) activated acetyl-CoA carboxylase 1 (*Acaca*) through decreasing the phosphorylation states of AMPK substrate sites on this protein^34^, thus increased the cellular level of malonyl-CoA which would act as a coupling factor in mediating insulin secretion^35^. As indicated by glucose time course data, prolonged exposure to glucose also induced phosphorylation change of extra targets involved in secretion pathway (Supplementary Fig. S8). Kinases activated or inhibited by glucose in a delayed manner, such as ERK, PKA and p38, indicated by the phosphorylation change of their own activation sites (Supplementary Fig. S3E), were more likely to be responsible for the phosphorylation dynamics of these new players (Supplementary Fig. S8).

### The kinome regulated by GLP-1 and glucose

**Figure 6:**
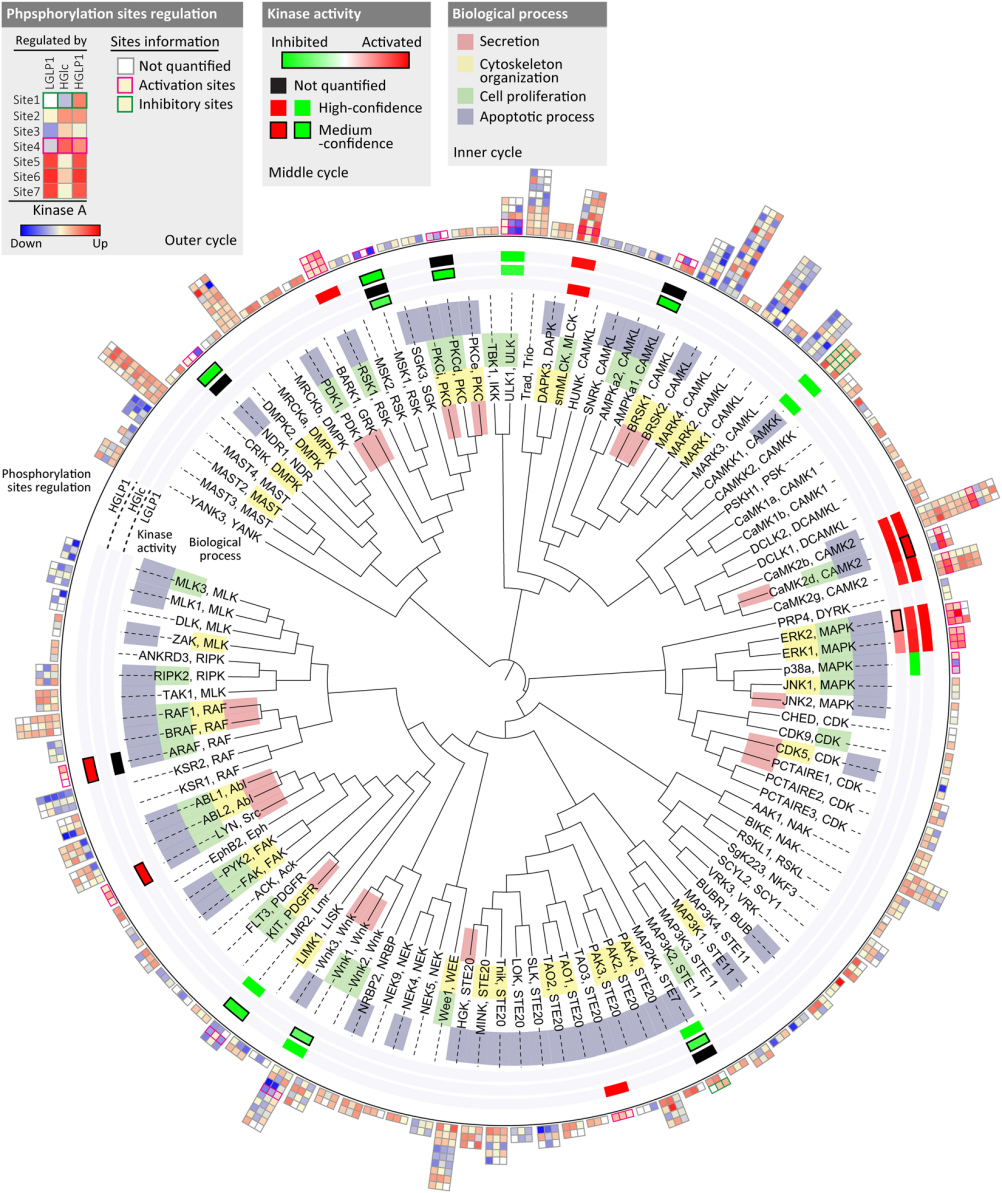
β-cell kinome regulated by GLP-1 and glucose. Regulated kinases were ordered by phylogenetic distance of the kinase domain retrieved from KinBase (mouse ortholog), and displayed using iTOL (see Methods for details). The phosphorylation change of each regulated sites on each kinase induced by different treatments were presented using heatmap in the outer rim. In the middle rim, the activation or inhibition status of regulated kinases were determined by the direction and amplitude of change of reported activation or inhibitory sites. The biological process that these kinases involved in were annotated according to GO database, and displayed in the inner rim. All proteins were listed by their corresponding gene names used by KinBase.

We quantified 1143 class 1 phosphorylation sites on more than 200 protein kinases, about half of the quantified β-cell kinome were regulated by GLP-1 (LGLP1), glucose (HGlc), or their synergy (HGLP1) (Supplementary Table S8). Regulated phosphorylation sites on these protein kinases are presented in Fig. 6, order on the basis of the similarity of their kinase domains^36^. The change of kinase activity were determined if the reported activation or inhibitory sites were regulated in this experiment, the annotation of biological processes they involved in were also included to see whether these regulated kinases were related to core β-cell function mediated by these secretagogues (Fig. 6). Kinase activity of AMPK, and its downstream substrate Unc51-like kinase 1 (ULK1), a key component involved in the autophagy process, were significantly inhibited by glucose, which would contribute to glucose induced β-cell survival, as well as insulin secretion^11, 37, 38^. PKA-dependent mechanism served an important part of regulation of β-cell function mediated by GLP-1, it also mediated part of GLP-1 action on β-cell kinome. Phosphorylation level of three PKA substrate sites (Ser74, Ser458, Ser475) on Ca^2+^/calmodulin-dependent protein kinase kinase α (CaMKK1) were increased after exposure to GLP-1, led to inhibition of this kinase^39, 40^. GLP-1 induced activation of myosin light chain kinase (MLCK) was also likely to proceed through a PKA-dependent mechanism, as the up-regulated activation sites on this kinase were also reported to be PKA substrate (Fig. 6)^41^. The regulation of these two kinases probably expanded the novel PKA-dependent pathway involved in GLP-1 action on pancreatic β-cells, as these two kinases were reported to function in regulating cell survival and hormone secretion^42, 43^. Several kinases were regulated by both GLP-1 and glucose, either on the same target sites or distinct sites within the identical kinase respectively (Supplementary Table S8). Although we did not observe p38 MAPK inhibition upon GLP-1 treatment as glucose did, its upstream kinase, mitogen-activated protein kinase kinase 4 (MAP2K4) was inhibited by both GLP-1 and glucose, via phosphorylating the identical reported inhibitory site (Fig. 6)^44^. Inhibition of MAP2K4 thus provided the possible common mechanism for both GLP-1 and glucose mediated suppression of β-cell apoptosis^44^. We also observed synergistic activation of ERK downstream protein kinase ribosomal protein S6 kinase alpha-1 (RSK1) mediated by GLP-1 plus glucose (HGLP1) (Fig. 6), through the actions of three reported activation sites on this kinase^45^. Glucose alone activated this kinase in a much delayed manner as indicated by glucose time course data (Supplementary Fig. S3E), co-administration of GLP-1 seemed to accelerate and potentiate the activation of p90S6K, thus contributed to facilitate β-cell survival.

## Discussion

Here, we applied global quantitative phosphoproteomics to explore the synergistic effect of GLP-1 and glucose in regulating INS-1E cell function at systems biology level. Our data localized more than 25,000 phosphorylation sites accurately on 5,389 proteins, generating the largest pancreatic β-cell phosphoproteome, and phosphorylation sites identified in previously published work^20^ were mostly included (Supplementary Fig. S3C). These data would serve as a resource to define actions of glucose and incretins in regulating β-cell function, such as insulin secretion and cell survival.

GLP-1 shares several common biological functions on pancreatic β-cells with glucose, such as inducing insulin secretion and cell survival^4^ and these two secretagogues also synergize in regulating several intracellular signaling^7^. The phosphorylation regulation mediated by the combination of these two secretagogues is mainly caused by addition of the effect mediated by GLP-1 and glucose alone at site level (Fig. 3B). We have found 128 phosphorylation sites on 123 proteins termed synergistic-regulated sites (Fig. 3A), co-administration of GLP-1 and glucose (HGLP1) leads the phosphorylation regulation in an additive manner towards these sites, and thus achieves the fold change threshold. Several phosphorylation sites are regulated by both glucose and GLP-1 (both-regulated sites defined in Fig. 1G), and proteins possessing both-regulated sites are enriched in signaling pathways highly related to β-cell function (Fig. 4D), such as insulin secretion pathway (Fig. 5). But for most of the both-regulated sites, synergistic regulation mediated by HGLP1 has not reached additive level (Fig. 3B, Supplementary Fig. S5A). It is likely that treatment of glucose or GLP-1 alone has already induced large proportion of phosphorylation, and the addition of the effect mediated by them would exceed full phosphorylation for these both-regulated sites. More extensively, GLP-1 and glucose implement their synergistic effect at pathway level when co-administration to cell (Fig. 4D). They prefer to regulate the same pathways which are highly related to β-cell functions but through several different targets, thus finally achieve the synergistic effect on β-cell functions at the pathway level.

The regulation of kinase activity mediated by GLP-1 and glucose was determined through both the overrepresentation of their predicted substrates and the actions of the activation or inhibitory sites on themselves (Fig. 2A, Fig. 6). We identified several novel kinases regulated by GLP-1 and glucose, and of course, not all regulated kinases were related to insulin secretion. Several kinases are regulated by both GLP-1 and glucose, such as PKA^46^, ERK^47^, and MAP2K4 which has not been reported. Inhibition of MAP2K4 would like to be part of the mechanism for both GLP-1 and glucose mediated suppression of β-cell apoptosis^44^.

Glucose controls the activity of distinct kinases in a strict temporal manner during the process of insulin secretion according to our glucose time course data. Several kinases, such as PKA, ERK and p38 are activated or inhibited by glucose in a delayed manner, indicated by the phosphorylation dynamics of their own activation site and their predicted or reported substrate sites (Supplementary Figs S3E and S8). It seems that several phosphorylation sites respond to GLP-1 more quickly than to glucose, such as substrates of PKA (Fig. 2B). Thus the synergy of GLP-1 with glucose not only results in activation of additional targets, but also in accelerating of kinase effect.

Through the dynamic regulation of the activity of kinases as well as protein phosphatase, the synergism of GLP-1 and glucose regulates insulin secretion in a complicated and exquisite way. For example, acute stimulation of glucose decreases the phosphorylation level of Ser541 on Kv2.1 (Fig. 5), possibly through Ca^2+^-dependent protein phosphatase calcineurin, thus activates this ion channel and leads to the repolarization of cell membrane^31^. While longer exposure to glucose inhibits the activity of p38, decreases the phosphorylation level of Ser804 on Kv2.1 which has been reported to be p38 substrate site (Supplementary Figs S3E and S8)^48^, thereby blocks the membrane insertion of this ion channel, finally decreases the outward K^+^ currents and maintained the duration of glucose-induced action potential for sustained phase of insulin secretory granule exocytosis^49^. Calcineurin has essential functions in β cells, such as inducing insulin gene transcription^50^, but glucose mediated activation of this protein phosphatase seems also to limit the secretion of insulin to a restricted level. However co-administration of GLP-1 would to some extent relieve this effect caused by glucose. Treatment of GLP-1 alone potentiates the phosphorylation level of the calcineurin target site (Ser541) on Kv2.1 (Fig. 5), thus prevented the possible inhibitory effect toward insulin secretion caused by glucose, and potentiates acute phase of insulin secretion.

Finally, we emphasized on the phosphorylation regulation of the secretion pathway, through both glucose/GLP-1 synergism data and glucose time course data (Fig. 5, Supplementary Fig. S8). The former delineated the synergistic regulatory network of secretion pathway mediated by glucose and incretins, and the latter would help us to determine the exact phase of insulin secretion these target sites involved in. Our data combined with technologies of examining the dynamic motion of secretory vesicles, such as total internal reflection fluorescence microscopy (TIRFM)^51^, would lead to the more comprehensive and deeper understanding of the dynamic regulatory network of the process of insulin secretion at systems-biology level.

## Methods

### Reagents and materials

GLP-1 7–36 amide, ^13^C_6_^15^N_2_-L-lysine, ^13^C_6_^15^N_4_-L-arginine, formaldehyde, ^13^C-D_2_-formaldehyde and cyanoborohydride were purchased from Sigma Aldrich (St. Louis, MO). Dialyzed heat-inactivated fetal bovine serum (FBS) was purchased from Biochrom AG (Merck, Germany). TiO_2_ beads were purchased from GL sciences (Tokyo, Japan).

### Cell culture

INS-1E cells were cultured in humidified atmosphere containing 5% CO_2_ in complete medium composed of RPMI1640 supplemented with 5% FBS, 1 mM sodium pyruvate, 50 μM 2-mercaptoethanol, 2 mM glutamine, 10 mM HEPES, 100 U/ml penicillin and 100 μg/ml streptomycin as previously described^52^. All INS-1E cells used in this study were between passages 60–80. For SILAC labeling in glucose time course experiment, medium were home-formulated with standard lysine and arginine replaced with ^13^C_6_^15^N_2_-L-lysine (40 mg/L) and ^13^C_6_^15^N_4_-L-arginine (25 mg/L, 1/10 of original amount). 200 mg/L standard L-proline was added to SILAC medium to eliminate arginine-proline conversion. Dialyzed FBS were supplemented into SILAC medium.

### Sample preparation

INS-1E cells were cultured in standard RPMI1640, before treatment INS-1E cells were washed and preincubated for 60 min at 37 °C in Krebs-Ringer bicarbonate HEPES buffer (KRBH, composition: 135 mM NaCl, 3.6 mM KCl, 5 mM NaHCO_3_, 0.5 mM NaH_2_PO_4_, 0.5 mM MgCl_2_, 1.5 mM CaCl_2_, and 10 mM HEPES, pH 7.4, BSA 0.1%) with 2.5 mM glucose. Next, after washing once with KRBH buffer containing 2.5 mM glucose, cells were incubated with 2.5 mM glucose KRBH buffer, or 2.5 mM glucose KRBH buffer with 10 nM GLP-1, or 16.7 mM glucose KRBH buffer, or 16.7 mM glucose KRBH buffer with 10 nM GLP-1 for 10 min, respectively. Then cells were lysed and harvested as described below. Forward and reverse stable isotope dimethyl labeling experiments were performed for each group of experiments, biological duplicates were analysed in both forward and reverse labeling experiments to evaluate the quantification repetitiveness of our experimental workflow.

### Protein digestion

After stimulation with indicated secretagogue for indicated time, INS-1E cell dishes were placed on ice, washed with cold PBS and lysed in SDT buffer (2% SDS, 0.1 M DTT, 0.1 M Tris/HCl, pH 7.6). After 3 min of heating at 95 °C and sonication, the samples were clarified by centrifugation for 10 min at 15,000 × g. Protein content was determined by measuring fluorescence at 295 nm for excitation and 350 nm for emission as described previously^53^. Filter-aided sample preparation protocol (FASP) using Amicon Ultra 15 Ultracel 30 k (Millipore) was applied for protein digestion. For dimethyl labeling-based proteomics experiment, 2 mg sample were digested with trypsin in 200 mM tetraethylammonium borohydride (TEAB), while for SILAC-based spike-in experiment, 4 mg of “light” sample plus 4 mg of “heavy” sample (internal standard) were mixed and digested with trypsin in 50 mM NH_4_HCO_3_ solution. For all these experiments, trypsin was added in a two rounds manner: 1/100 of total protein amount for the first round, digested for 12 hours, and then 1/150 of total protein amount for the second round, digested for additional 5 hours. The resulting peptides were collected through centrifugation.

### Stable isotope dimethyl labeling of peptides

For GLP-1/glucose synergism experiments, resulting peptides from digestion were stable isotope dimethyl labeled as described previously^54^. Briefly, in forward labeling replicates, peptides of condition A (Fig. 1A) were labeled with a mixture of formaldehyde-H_2_ (CH_2_O) and cyanoborohydride (NaBH_3_CN) (“light” reagent), peptides of condition B were labeled with ^13^C-D_2_- formaldehyde (^13^CD_2_O) and cyanoborohydride (NaBH_3_CN) (“heavy” reagent). In reverse labeling replicates, reagents for labeling samples from condition A and condition B were swapped. After quenching by adding ammonia solution and formic acid, the “light” and “heavy” dimethyl-labeled samples were mixed in 1:1 ratio based on total peptide amount determined by measuring fluorescence as described above.

### Phosphopeptide enrichment and fractionation

2 mg of total peptides (1 mg “light” sample + 1 mg “heavy” sample) were needed for phosphopeptide enrichment, and strong cation exchange (SCX) method was used for subsequent phosphopeptide fractionation. Phosphopeptide enrichment by TiO_2_ was performed as previously described^55^. Briefly, 0.5 mg of mixed peptides were incubated with 1 mg of TiO_2_ beads and rotated for 10 minutes, and then this slurry was transferred to Empore-C_8_ StageTips. After three washes, bound peptides were eluted using 1% NH_4_OH in 40% acetonitrile. 4 aliquots of phosphopeptide elute were mixed together and vacuum dried for subsequent SCX fractionation. Phosphopeptides were dissolved in low pH buffer (10 mM malonic acid, 214 μL formic acid, titrated with trifluoroacetic acid to pH 2.0) and loaded onto home-packed SCX column, a linear pH gradient was performed on Agilent 1100 HPLC by increasing the proportion of high pH buffer (10 mM malonic acid, 214 μL formic acid, titrated with ammonia solution to pH 9.0) to 100%, phosphopeptides elute were automatically collected and combined into six fractions. Each fraction should be desalted before LC-MS/MS analysis.

### Mass spectrometry

Eluted phosphopeptides were separated through a nano-emitter column (15 cm length, 75 μM inner diameter) packed in-house with ReproSil-Pur 3-μm C_18_-AQ resin (Dr. Maisch GmbH) and introduced into mass spectrometry via a nanoelectrospray ion source (source voltage, 1.9–2.2 kV). A linear gradient from 4% to 30% buffer B (buffer A, 0.1% formic acid in ddH2O; buffer B, 0.1% formic acid in acetonitrile) over 150 min was used for peptide separation at a flow rate of 250 nL/min.

For collision-induced dissociation (CID) mode performed on LTQ-Orbitrap XL (Thermo Fisher Scientific), a full scan was acquired at a target value of 1,000,000 ions with resolution R = 60,000 at *m/z* 400. The six most intense ions were sequentially isolated with a window of 2 Th for MS/MS sequencing, the multistage activation algorithm was enabled using the neutral loss values of 97.97, 48.99, 32.66 and 24.49 m/z units to improve the fragmentation of phosphopeptides. Higher energy collisional dissociation (HCD) was performed on Q-Exactive (Thermo Fisher Scientific), with a resolution of 70,000 at *m/z* 400 for full scan and 17,500 for MS/MS scans. Top twelve ions were selected at an isolation window of 2.0 *m/z* units and accumulated to a target value of 2e5 for MS/MS sequencing. Dynamic exclusion was enabled to void choosing former target ions for 180 s, and lock-mass was enabled using 445.120025.

### Mass spectrometric data analysis by MaxQuant

Raw mass spectrometry data was processed using the MaxQuant software^56^ version 1.3.0.5 using the default settings with minor changes: Oxidation (Methionine), Acetylation (Protein N-term) and Phospho (STY) were selected as variable modifications, and Carbamidomethyl (C) as fixed modification. Enzyme specificity was set to trypsin, additionally allowing cleavage N-terminal to proline and up to two missed cleavages. For spike-in SILAC experiments, Arg0/Lys0 was selected as light labels and Arg10/Lys8 was selected as heavy labels; for dimethyl-labeling experiments, DimethLys0/DimethNter0 was selected as light labels and DimethyLys6/DimethNter6 was selected as heavy labels. Database searching was performed using the Andromeda search engine^57^ against the Rat IPI database v3.77 (39,495 protein entries) concatenated with known contaminants and reversed sequences of all entries. Peptide identification was based on a search with an initial mass deviation of the precursor ion of up to 6 ppm, and the allowed fragment mass deviation was set to 20 ppm for HCD and 0.5 Da for CID fragmentation. A false discovery rate of less than 0.01 for proteins, peptides, and phosphosites and a minimum peptide length of 6 amino acids were required. Phosphorylation sites were assigned as previously described^58^. Quantification of SILAC pairs was performed by MaxQuant with standard settings. The “match between runs” option was enabled with a time window of 1 min to transfer identifications in the same fraction, only for samples analyzed using the same chromatography and nanospray conditions.

### Determination of regulated sites in GLP-1/glucose synergism data set

For each experiment (Exp1, Exp2 and Exp3 in Fig. 1A), phosphorylation sites were quantified using the ratio from forward and reverse labeling replicates which used the least Nmods phosphopeptides to quantify the corresponding sites. Regulated sites were classified into two categories. High-confidence regulated sites were defined if the ratio condition B/A reached the 1.75-fold change threshold (determined by boxplot algorithm) in both forward and reverse labeling replicates. Phosphorylation sites satisfied with one of the following two criterions were determined as medium-confidence regulated sites: (i), if phosphorylation sites were quantified in only one of the forward and reverse labeling replicates (sites quantified with different Nmods phosphopeptides in forward and reverse replicates also belonged to this situation), the ratio condition B/A should reach the 1.75-fold change threshold in the adopted quantified experiment; (ii), if phosphorylation sites were quantified in both forward and reverse labeling replicates with the same Nmods phosphopeptides, the ratio condition B/A should reach the 1.75-fold change threshold in one replicate and 1.5-fold in the other. Regulate sites, both high-confidence and medium-confidence, should be identified with MS/MS spectra in at least one of the adopted quantified experiments.

### Analysis of kinase substrates overrepresentation

Kinase-substrate relationships were predicted using NetworKIN 3.0^22^ for all class 1 sites identified. Predicted kinases were filtered using NetworKIN scores > 1.4 and Max difference < 4 for significance. High confidence regulated sites were clustered according to their predicted kinase and compared to quantified sites for kinase substrates enrichment. Only kinases with *P* value < 0.05 (Hypergeometric test, p-value was adjusted by Benjamini & Hochberg algorithm) were kept, and filtered *P* value matrix was transformed by the function x = −log_10_(*P* value). This matrix was then scaled and presented in heatmap. Over- and underrepresented kinase predictions were colored yellow and blue, respectively (Fig. 2A).

### Sequence homology analysis of kinases

Clustering of regulated kinases according to their kinase domain homology was performed as previously described^21^. FASTA sequences of the kinase domain of all regulated kinases were extracted from KinBase, using mouse ortholog, then these sequences were aligned by ClustalX2^59^ using the default parameters. For visualization of the alignment, a phylogenetic tree was calculated with the neighbor-joining algorithm, exported in Phylip format, and uploaded to the Interactive Tree of Life (iTOL)^60^. The change of phosphorylation status of each regulated site and the activity alteration of each kinase were presented in this phylogenetic tree. The biological processes these kinases involved in were annotated by Gene Ontology (GO) database^61^, and also displayed in this phylogenetic tree.

### KEGG pathways enrichment

KEGG pathway enrichment analysis was performed by comparing proteins with high-confidence regulated sites with proteins having phosphosites quantified in both forward and reverse labeling replicates to select pathways intensively regulated by the indicated treatment. DAVID algorithm^62, 63^ was used for KEGG pathway enrichment analysis with default parameters.

## Electronic supplementary material

Supplementary information

Supplementary Table S1

Supplementary Table S2

Supplementary Table S3

Supplementary Table S4

Supplementary Table S5

Supplementary Table S6

Supplementary Table S7

Supplementary Table S8
