# Supplementary material for: Systematic Synergy of Glucose and GLP-1 to Stimulate Insulin Secretion Revealed by Quantitative Phosphoproteomics
Source: Sci Rep. 2017 Apr 21;7:1018. doi: 10.1038/s41598-017-00841-1 (PMC5430885; doi:10.1038/s41598-017-00841-1)
Supplement: Supplementary file 1 — Supplementary information [file 41598_2017_841_MOESM1_ESM.pdf]

Supplementary Information

Systematic Synergy of Glucose and GLP-1 to Stimulate Insulin Secretion Revealed by Quantitative Phosphoproteomics

Jia-shu Tang[1], Qing-run Li[1], Jia-ming Li[1], Jia-rui Wu[1,2*], Rong Zeng[1,2*]

[1]Key Laboratory of Systems Biology, Institute of Biochemistry and Cell Biology, Shanghai Institutes for Biological Sciences, Chinese Academy of Sciences, 320 Yue-Yang Road, Shanghai, 200031, China

[2] Department of Life Sciences, ShanghaiTech University, 99 Haike Road, Shanghai, 201210, China

The supplementary information contains three sections:

I. Supplementary Methods

Sample preparation for glucose time course experiments

Strong anion exchange fractionation of phosphopeptides

Multiple TiO_2 incubation strategy for phosphopeptide enrichment

Determination of glucose regulated phosphorylation sites in glucose time course data set

Measurement of insulin secretion

II. Supplementary Figures

Figure S1. General properties of our phosphoproteomics data set.

Figure S2. Definition of high confidence and medium confidence regulated sites.

Figure S3. Glucose time course experiments.

Figure S4. Definition of ratio difference rate from additive effect.

Figure S5. Regulation pattern mediated by GLP-1 and glucose towards both-regulated sites.

Figure S6. Phosphoproteins regulated by both glucose and GLP-1.

Figure S7. KEGG pathway enrichment analysis for proteins regulated by GLP-1 (LGLP1), glucose (HGlc) and their synergism (HGLP1).

Figure S8. Glucose mediated temporal regulation of insulin secretory granule-associated proteins.

III. Supplementary Tables

Table S1. All identified phosphorylation sites in glucose/GLP-1 synergism experiments.

Table S2. All regulated phosphorylation sites in glucose/GLP-1 synergism experiments.

Table S3. All identified phosphorylation sites in glucose time course experiments.

Table S4. All regulated phosphorylation sites in glucose time course experiments.

Table S5. Comparison of glucose and GLP-1 regulated phosphorylation sites.

Table S6. HGLP1 high confidence regulated sites for evaluation of synergistic effect mediated by GLP-1 and glucose at site level.

Table S7. Secretion pathway regulated by glucose and GLP-1 synergistically.

Table S8. All identified class 1 phosphorylation sites of β-cell kinome.

Supplementary methods

Sample preparation for glucose time course experiments

“Heavy” labeled INS-1E cells were cultured in SILAC medium for nine cell doublings, labeling efficiencies were determined by mass spectrometry and were found to be > 97%. When reaching near 90% confluence, INS-1E cells were washed and preincubated for 60min at 37℃ in KRBH buffer with 2.5 mM glucose. Next, after washing once with KRBH buffer containing 2.5 mM glucose, cells were then incubated in KRBH buffer containing 16.7 mM glucose for 0 min, 2 min, 8 min and 30 min each. Incubation was stopped by placing the dishes on ices, cells were harvested as mentioned above. “Heavy” labeled cell lysates from each glucose treatment time point were mixed together to generate a “heavy” internal standard pool. “Light” labeled cells were cultured in standard RPMI1640 to desired population, cell lysates from each glucose treatment time point were obtained as described above. Same amount of each “light” protein sample were mixed with “heavy” internal standard, and then proceeded to protein digestion, phosphopeptides enrichment and LC-MS/MS analysis together. Each time point treatment experiments were performed in three biological replicates.

Strong anion exchange fractionation of phosphopeptides

Protein digest from 8 mg total protein (4 mg “light” sample + 4mg “heavy” sample) was needed for phosphopeptide enrichment if strong anion exchange (SAX) method was used for subsequent phosphopeptide fractionation. In this case, phosphopeptide from protein digest of 2 mg total protein were enriched using 2 mg of TiO_2 beads, 4 aliquots of phosphopeptides elute were mixed together and vacuum dried for subsequent SAX fractionation [1] as

previously described. Briefly, Britton & Robinson buffer (20 mM CH_3COOH, 20mM H_3PO_4, 20 mM H_3BO_3) were titrated with NaOH to pH 11, 8, 6, 5, 4, 2, these series of solution were used for stepped elution of phosphopeptide loaded onto StageTip-based SAX microcolumn. Phosphopeptide elute of each fraction should be desalted before LC-MS/MS analysis.

Multiple TiO_2 incubation strategy for phosphopeptide enrichment

Multiple TiO_2 incubation procedure for phosphopeptide enrichment was performed as previously described with slight modifications [2]. Protein digest from 4 mg of total protein (2 mg "light" sample + 2 mg "heavy" sample) was dissolved in solution containing 2% TFA (v/v), 30% ACN (v/v). 5 μl TiO_2 slurry containing 0.5 mg TiO_2 beads were added to each sample and rotated for 5 min. After centrifugation for 2 min at 6,000 × g, the supernatant was transferred to a new tube and a new aliquot of 5 μl TiO_2 slurry was added for the second round of incubation. This step should be repeated eight times to generate eight fractions, then the phosphopeptides bound on TiO_2 beads in each fraction were eluted separately as described above. Each fraction of phosphopeptide was then analysed by LC-MS/MS apart.

Determination of glucose regulated phosphorylation sites in glucose time course data set

Phosphorylation sites identified were considered as glucose regulated if they met the criterions below: (i), phosphopeptides with the same number of phosphorylation sites (Nmods in MaxQuant) were used to quantify the site identified in both glucose treatment time point and basal condition; (ii), phosphorylation site should be identified with MS/MS spectra in both glucose treatment time point and basal condition; (iii), the ratio of glucose treatment time point to basal condition should achieve 1.5 fold change threshold (determined by boxplot algorithm).

Measurement of insulin secretion

INS-1E cells were preincubated for 60min at 37℃ in KRBH buffer containing 2.5mM glucose. The cells were then incubated in KRBH buffer supplemented with indicated stimuli for 30min or 60min. The KRBH buffer media were then collected and insulin content was measured by rat insulin ELISA kit (Mercodia, Sweden).

Supplementary Figure S1

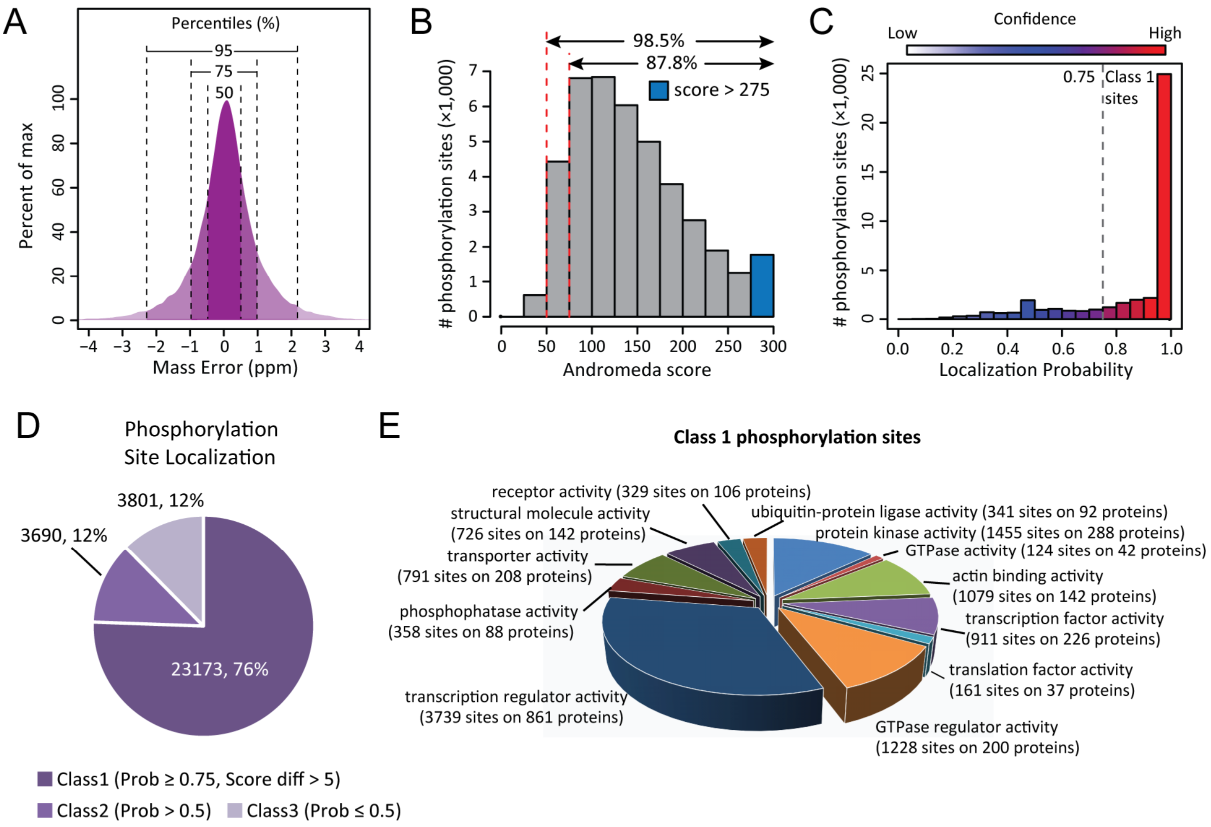

Supplementary Figure S1. General properties of our phosphoproteomics data set (related to Fig 1). (A) Distribution of measured mass error for all sequenced phospho-peptides. **(B-C)** Distribution of database search engine (Andromeda) scores **(B)** and localization probability **(C)** for all phosphorylation sites identified. **(D)** Distribution of all class 1, class 2, and class 3 sites. **(E)** GO molecular function annotation of proteins identified with class 1 phosphorylation sites.

Supplementary Figure S2

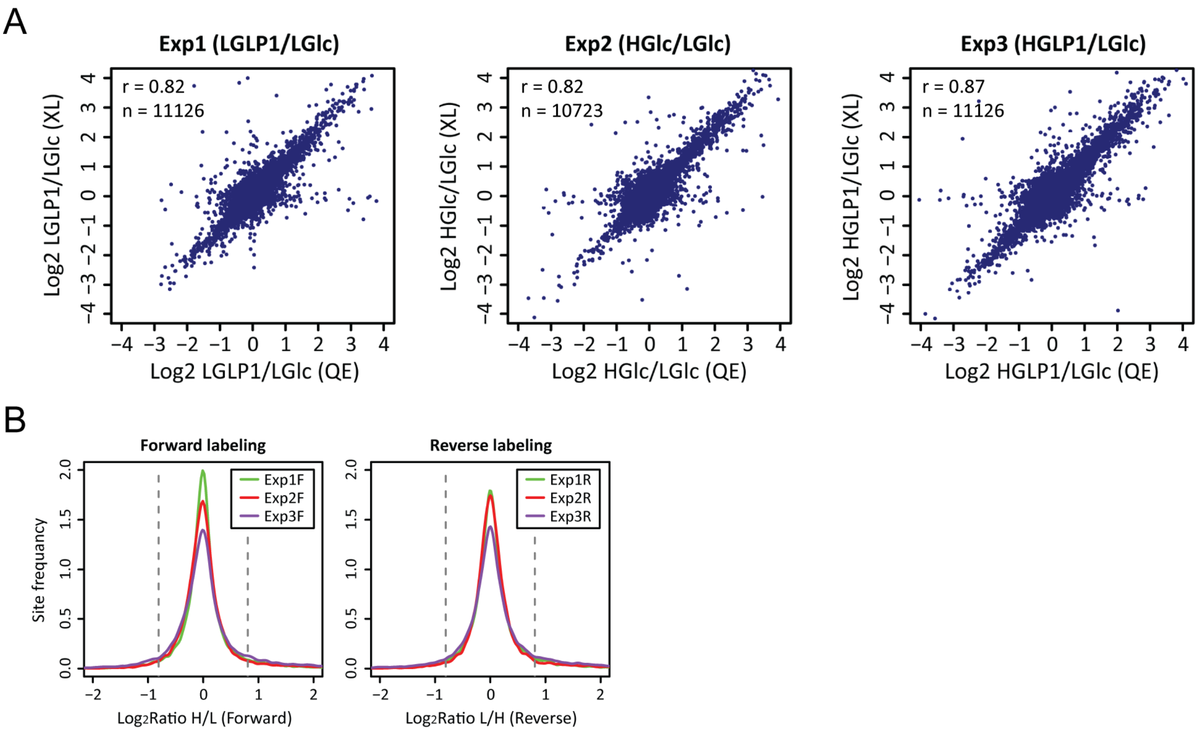

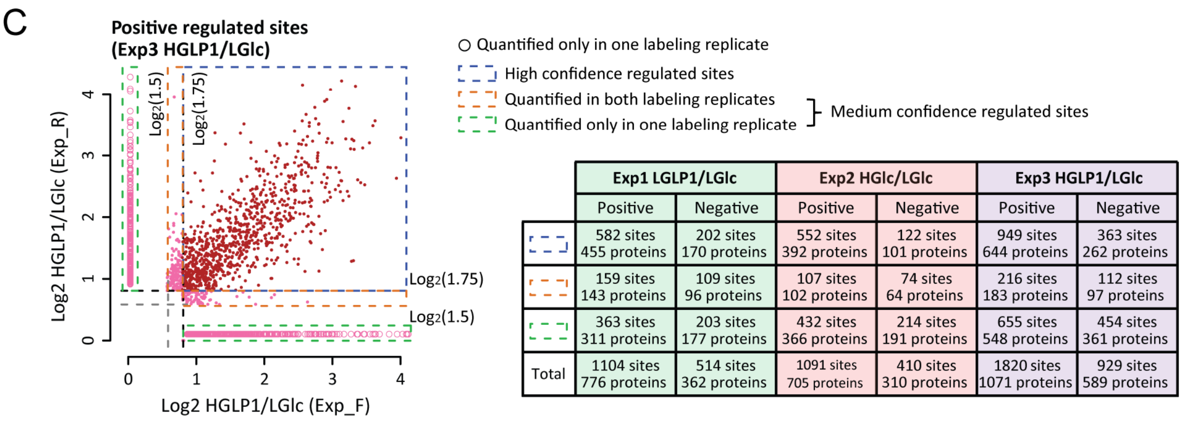

	Exp1 LGLP1/LGlc		Exp2 HGlc/LGlc		Exp3 HGLP1/LGlc	
	Positive	Negative	Positive	Negative	Positive	Negative
High confidence regulated sites (blue)	582 sites<br>455 proteins	202 sites<br>170 proteins	552 sites<br>392 proteins	122 sites<br>101 proteins	949 sites<br>644 proteins	363 sites<br>262 proteins
Quantified in both labeling replicates (orange)	159 sites<br>143 proteins	109 sites<br>96 proteins	107 sites<br>102 proteins	74 sites<br>64 proteins	216 sites<br>183 proteins	112 sites<br>97 proteins
Quantified only in one labeling replicate (green)	363 sites<br>311 proteins	203 sites<br>177 proteins	432 sites<br>366 proteins	214 sites<br>191 proteins	655 sites<br>548 proteins	454 sites<br>361 proteins
Total	1104 sites<br>776 proteins	514 sites<br>362 proteins	1091 sites<br>705 proteins	410 sites<br>310 proteins	1820 sites<br>1071 proteins	929 sites<br>589 proteins

Supplementary Figure S2. Definition of high confidence and medium confidence regulated sites. (A) Quantitative reproducibility for biological replicates in all three experiments. Ratios from forward or reverse labeling replicates in the same treatment experiments are plotted in the same chart. **(B)** Distribution of ratio condition B/A in all three experiments. Forward and reverse labeling experiments are calculated apart. Dashed line indicates 1.75-fold change threshold. **(C)** Determination of medium-confidence regulated sites. Schematic diagram of high-confidence and medium-confidence regulated sites was exemplified using positive-regulated sites in Exp3 (HGLP1/LGlc) (left). High-confidence and medium-confidence regulated class 1 sites across three experiments were counted (right).

Supplementary Figure S3

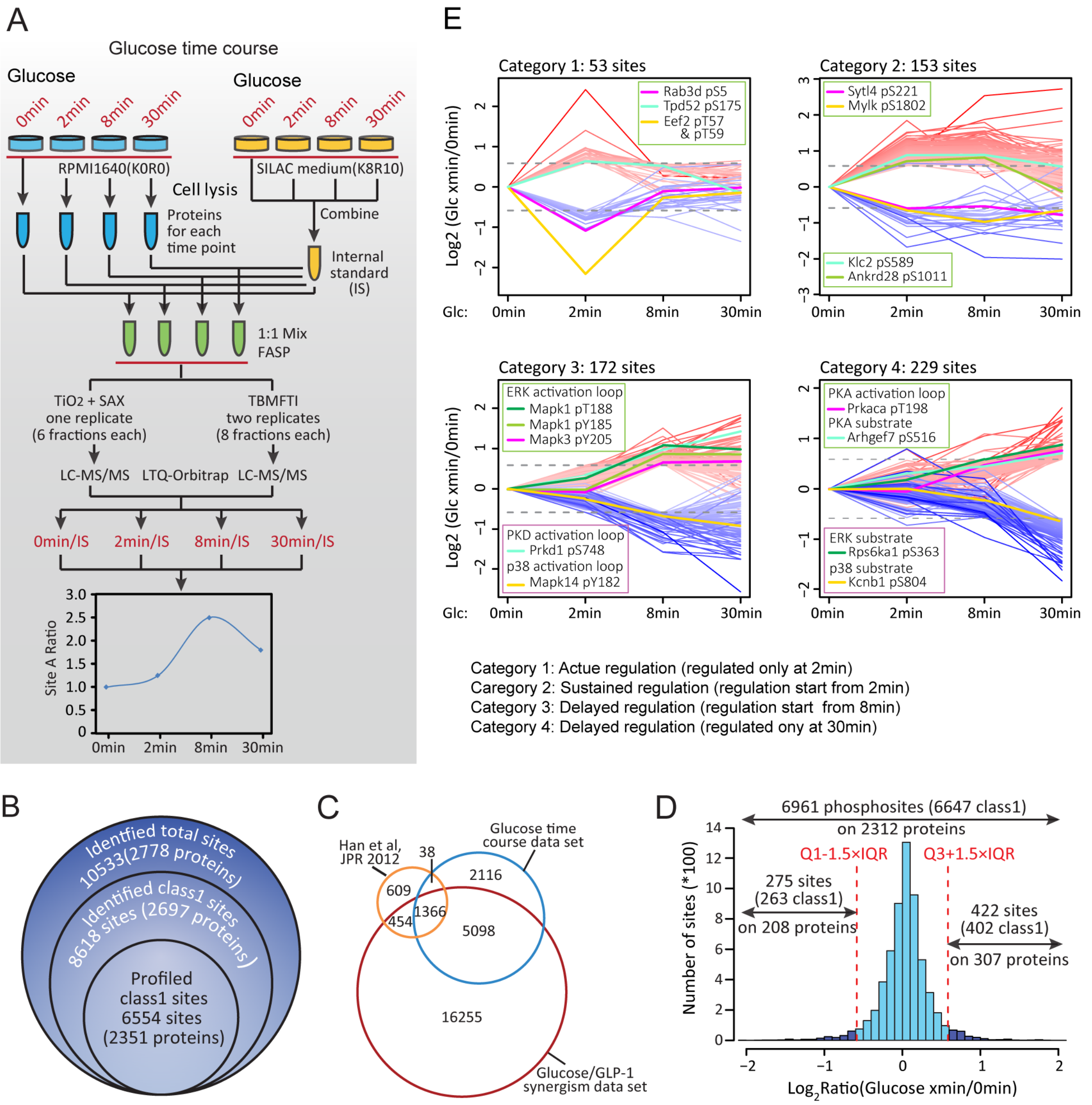

Supplementary Figure S3. Glucose time course experiments. (A) Workflow diagram of the glucose time course experiment. SILAC-based spike-in strategy was applied for the quantitative phosphoproteomic study. Three biological replicates were performed for each time point treatment. **(B)** Summary of the identified and quantified β-cell phosphoproteome in glucose time course experiment. **(C)** Venn-diagram showing the overlap of identified class 1 sites among glucose time course dataset, glucose/GLP-1 synergism dataset and other literature reported β-cell phosphoproteome. **(D)** Boxplot algorithm to screen glucose regulated phosphorylation sites (purple). **(E)** Glucose regulated sites were classified into four categories according to different stages of GSIS. Shaded red lines stand for the

positive-regulated sites, shaded blue lines standard for the negative-regulated sites. Colored lines represent some example sites with their biological functions reported, dashed gray line indicates the fold change threshold (1.5 in this data set). Glc is short for glucose.

Supplementary Figure S4

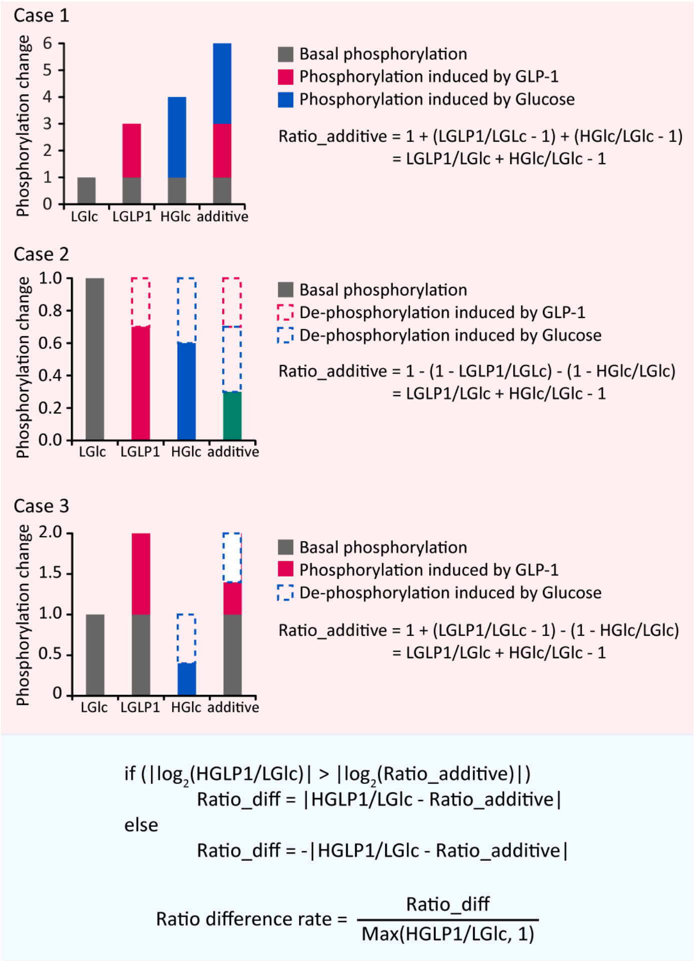

Supplementary Figure S4. Definition of ratio difference rate from additive effect. This rate was defined to evaluate whether GLP-1 and glucose induced the change of phosphorylation status in an additive manner. Ratio difference rate equals zero means GLP-1 and glucose behave in exactly the additive manner.

Supplementary Figure S5

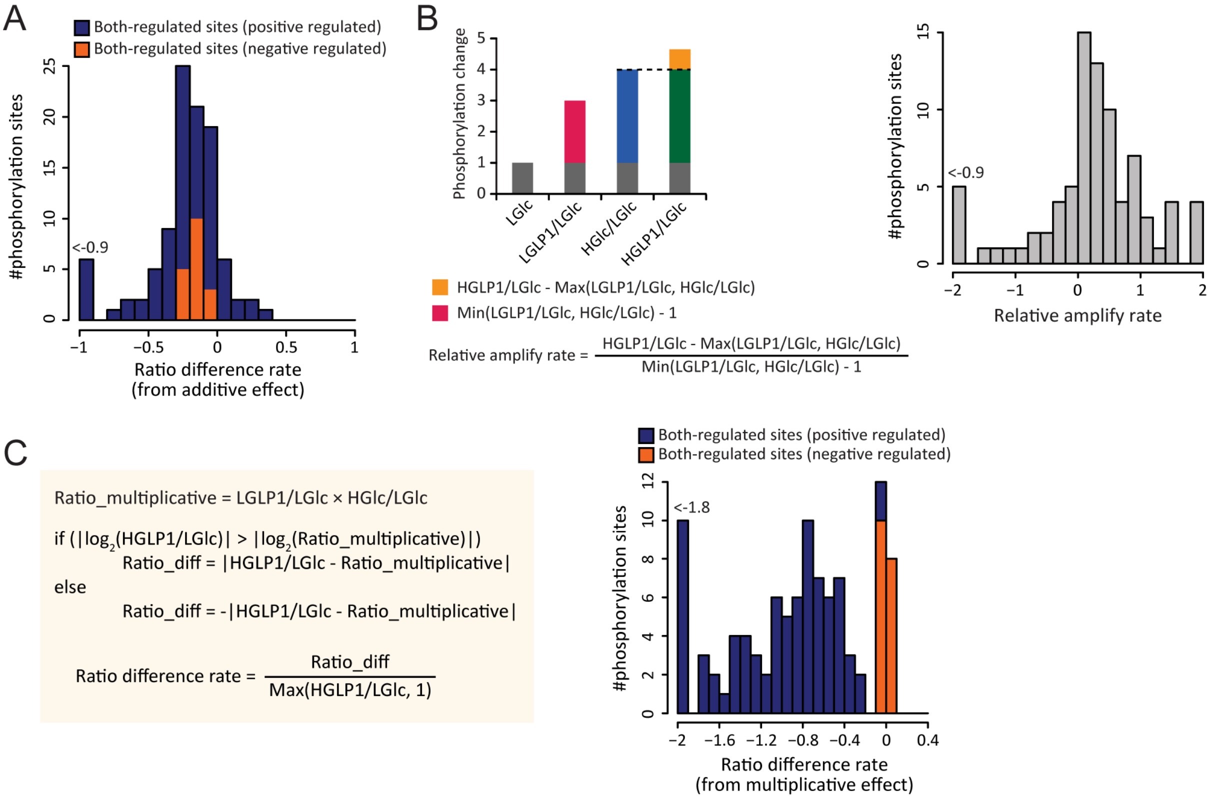

Supplementary Figure S5. Regulation pattern mediated by GLP-1 and glucose towards both-regulated sites (related to Fig 3). (A) Distribution of the ratio difference rate from additive effect for both-regulated sites. Positively and negatively regulated sites were calculated separately. **(B)** Distribution of the relative amplify rate for phosphorylation sites positively regulated by both GLP-1 and glucose (both-regulated sites). This rate was defined to evaluate whether the co-operation of GLP-1 and glucose (HGLP1) can induce more significant phosphorylation change for a single site. The definition of relative amplify rate is listed on the left. **(C)** Distribution of the ratio difference rate from multiplicative effect for both-regulated sites. The definition of ratio difference rate from multiplicative effect is listed on the left.

Supplementary Figure S6

A

	Phosphoproteins regulated by GLP-1	Phosphoproteins quantified in GLP-1 treatment
Phosphoproteins regulated by Glucose	317 proteins	702 proteins
Phosphoproteins quantified in Glucose treatment	838 proteins	4187 proteins

P value = 1.15e-64; Hypergeometric testing
Odds ratio = 2.26

B

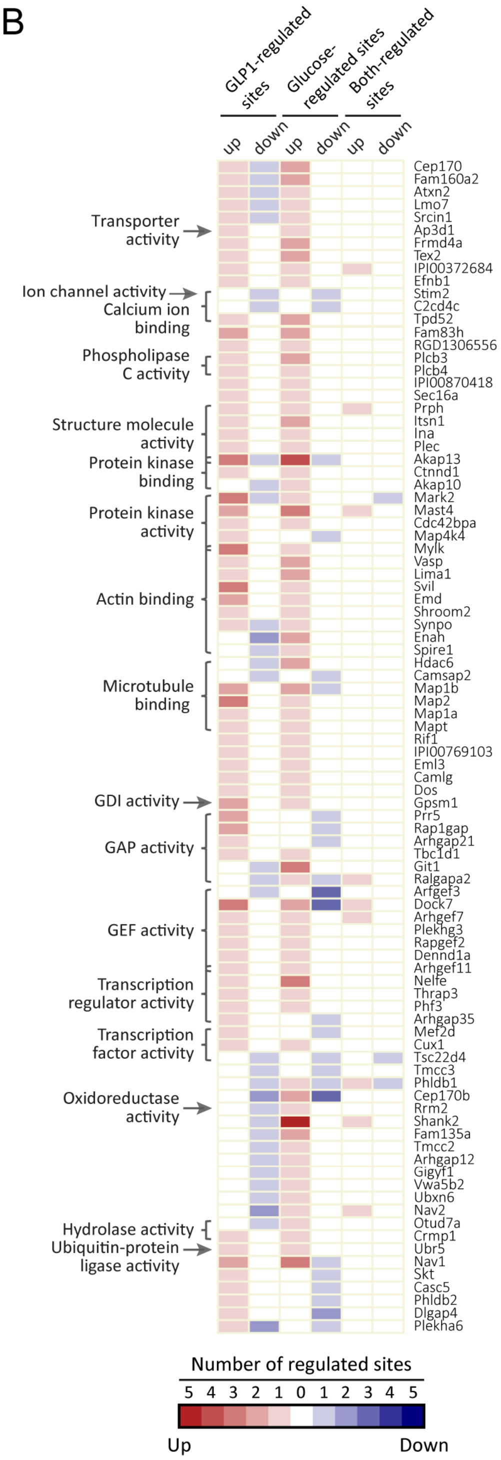

Supplementary Figure S6. Phosphoproteins regulated by both glucose and GLP-1. (A) Fourfold table showing the number of phosphoproteins regulated by GLP-1 (LGLP1) and glucose (HGlc). Hypergeometric test was performed to evaluate whether these two secretagogues tend to regulate the same protein. Phosphoproteins with both high-confidence and medium-confidence regulated sites

were taken into count. **(B)** Heatmap showing the number of phosphorylation sites with different regulation patterns (defined in Fig 1G) on each protein to clarify synergistic regulation at protein level. Only phosphoproteins with high confidence HGLP1 regulated sites that were regulated by both GLP-1 (LGLP1) and glucose (HGlc), but at distinct phosphorylation sites were presented. The molecular function of each phosphoprotein annotated by GO database were listed.

Supplementary Figure S7

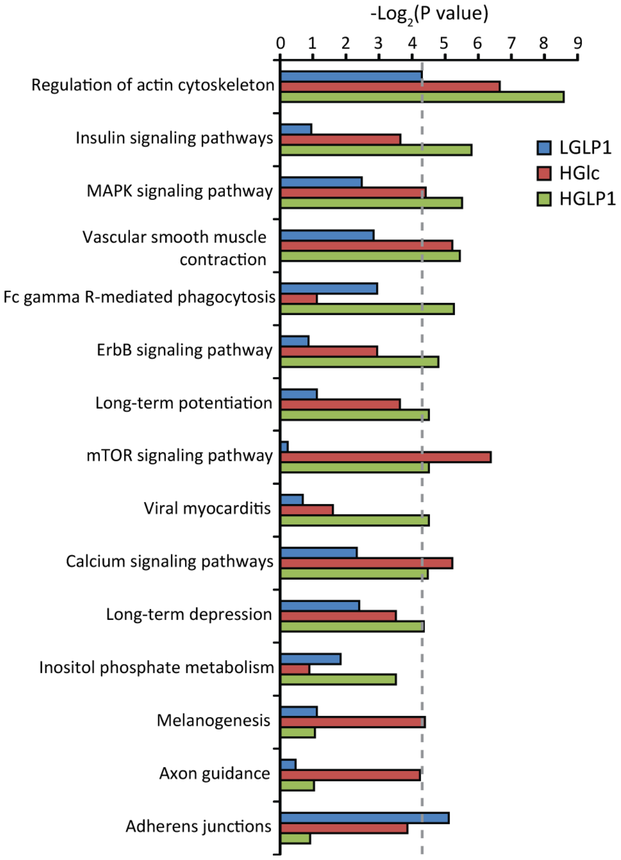

Supplementary Figure S7. KEGG pathway enrichment analysis for proteins regulated by GLP-1 (LGLP1), glucose (HGlc) and their synergism (HGLP1). Proteins with phosphorylation sites regulated by LGLP1, HGlc or HGLP1 with high confidence were analysed by DAVID algorithm for KEGG pathway enrichment. Dashed gray line means *P* value (Benjamini & Hochberg algorithm adjusted) equals 0.05.

Supplementary Figure S8

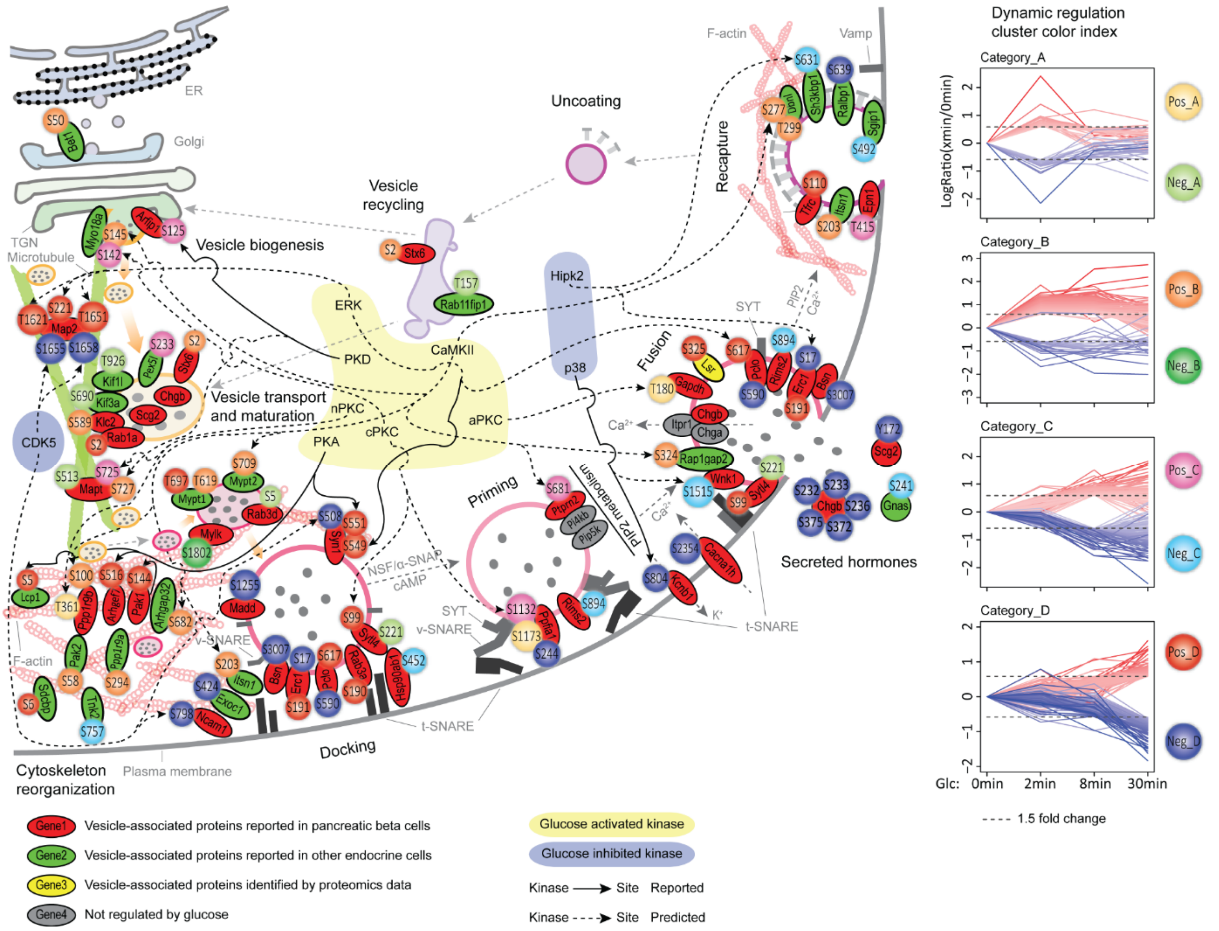

Supplementary Figure S8. Glucose mediated temporal regulation of insulin secretory granule-associated proteins. Insulin secretory granule-associated proteins (ISG proteins) were annotated by GO database or previously published proteomics data [3]. ISG proteins regulated in glucose time course data were exhibited with respect to their subcellular localization and the steps of the exocytosis pathway they involved in. The regulated sites on these proteins were color coded according to the temporal phosphorylation change patterns as shown in Supplementary Fig. S3E. The kinase-substrate relationship was also shown, either reported by literature or predicted by NetworKIN. Pos is short for positive-regulated; Neg is short for negative-regulated.

Supplementary References

1 Wisniewski, J. R., Nagaraj, N., Zougman, A., Gnad, F. & Mann, M. Brain phosphoproteome obtained by a FASP-based method reveals plasma membrane protein topology. *Journal of proteome research* 9, 3280-3289, doi:10.1021/pr1002214 (2010).

2 Zanivan, S. *et al.* Solid tumor proteome and phosphoproteome analysis by high resolution mass spectrometry. *Journal of proteome research* 7, 5314-5326 (2008).

3 Brunner, Y. *et al.* Proteomics analysis of insulin secretory granules. *Molecular & cellular proteomics : MCP* 6, 1007-1017, doi:10.1074/mcp.M600443-MCP200 (2007).
